# Subterranean Microbiome Affiliations of Plantain (*Musa* spp.) Under Diverse Agroecologies of Western and Central Africa

**DOI:** 10.1007/s00248-021-01873-x

**Published:** 2021-09-29

**Authors:** Manoj Kaushal, Yao Kolombia, Amos Emitati Alakonya, Apollin Fotso Kuate, Alejandro Ortega-Beltran, Delphine Amah, Cargele Masso

**Affiliations:** 1 International Institute of Tropical Agriculture (IITA), Mikocheni B, Dar es Salaam, Tanzania; 2 International Institute of Tropical Agriculture (IITA), Oyo Road, PMB 5320, Ibadan, 200001 Nigeria; 3 International Maize and Wheat Improvement Center (CIMMYT), México-Veracruz, El Batán Km. 45, 56237 Texcoco, Mexico; 4 International Institute of Tropical Agriculture (IITA), BP 2008 (Messa), Yaounde, Cameroon

**Keywords:** Microbial diversity, *Musa* spp., Agroecologies, Smallholder farmers, Metagenomics

## Abstract

Plantain (*Musa* spp.) is a staple food crop and an important source of income for millions of smallholder farmers in sub-Saharan Africa (SSA). However, there is a paucity of knowledge on soil microbial diversity in agroecologies where plantains are grown. Microbial diversity that increases plant performance with multi-trophic interactions involving resiliency to environmental constraints is greatly needed. For this purpose, the bacterial and fungal communities of plantain fields in high rainfall forests (HR) and derived savannas (SV) were studied using Illumina MiSeq for 16S rDNA and ITS amplicon deep sequencing. Microbial richness (α- and β-diversity), operational taxonomic units, and Simpson and Shannon–Wiener indexes (observed species (Sobs), Chao, ACE; *P* < 0.05) suggested that there were significant differences between HR and SV agroecologies among the most abundant bacterial communities, and some specific dynamic response observed from fungal communities. Proteobacteria formed the predominant bacterial phylum (43.7%) succeeded by Firmicutes (24.7%), and Bacteroidetes (17.6%). Ascomycota, Basidiomycota, and Zygomycota were the three most dominant fungal phyla in both agroecologies. The results also revealed an immense array of beneficial microbes in the roots and rhizosphere of plantain, including *Acinetobacter*, *Bacillus*, and *Pseudomonas* spp. COG and KEGG Orthology database depicted significant variations in the functional attributes of microbes found in the rhizosphere to roots. This result indicates that the different agroecologies and host habitats differentially support the dynamic microbial profile and that helps in altering the structure in the rhizosphere zone for the sake of promoting synergistic host-microbe interactions particularly under resource-poor conditions of SSA.

**Supplementary Information:**

The online version contains supplementary material available at 10.1007/s00248-021-01873-x.

## Introduction

*Musa* spp. (banana and plantain) are a key source of income and an essential commodity in international and local trade in many developing countries of South America, South-East Asia, and Africa. In sub-Saharan Africa (SSA), banana and plantain are considered as major staple food crop supporting the livelihoods of millions of smallholder farmers. For instance, in 2018, 27% of the total global banana production occurred in SSA with most of it from small farms and backyard gardens [1]. Because plantain plants produce fruits throughout the year, they serve as an important food security crop and a source of income for 70 million people in the west and central parts of SSA. Considering the demographic projections, low plantain yields (4–10 t/ha/year depending on plantation density) and high demands accompanied with increased pressure of the human population are likely to worsen the future of food security in the region. The constant infestation by many pests and diseases cause severe damage to the crop resulting in low productivity [2, 3]. Also, studies on the biotic and abiotic constraints revealed that humid tropics are the most favorable for plantain production [4, 5].

Given the potential of plantain as a source of food and income and its role in poverty alleviation, there is a need for policies and technologies that will stimulate its production and productivity in SSA. The development of the genetic variants through breeding approaches to overcome various abiotic and biotic constraints is a slow and complex process. In addition, controlling pests and diseases using synthetic chemicals, and under climatic variations, is difficult and out of the option for smallholder farmers due to associated costs. Therefore, overcoming these obstacles and balancing plantain production require highly sustainable and cost-effective strategies to contribute to food security in SSA.

For decades, soil microbes have been considered as key for protecting numerous crops from various biotic and abiotic constrains. It is well documented that the increase in beneficial microbial diversity of soil can allow controlling various soil-borne diseases and preventing the establishment of injurious pathogens in the rhizosphere and roots of a host plant [6, 7]. For *Musa* spp., cropping practices tend to influence microbial community structures and compositions and these vary under diverse agroecologies and climatic conditions [8, 9]. In reverse, beneficial microbial communities associating with a crop may provide excellent health and optimal production by inhibition of pathogens and subsequent disease [10]. Common biocontrol agents include *Pseudomonas* spp., *Bacillus* spp., *Sphingobium* spp., and *Chaetomium* spp. [11, 12]. These biocontrol agents drive the suppression of pathogenic microbes which are further enhanced by the combined antimicrobial actions exerted among beneficial and pathogenic microbes. Soil properties, such as pH, influence structures and compositions of microbial communities and their relative abundance, hence may contribute to general disease suppression/control [13]. Promoting soil health is another important component that needs to be addressed for enhanced biological and microbial diversity in soil. Soil health could be improved by applying organic amendments that aim to upgrade above- and below-ground biodiversity, thereby stimulating soil microbial biomass and activity [14–16]. Apart from the protection against pests and diseases and raising soil health, beneficial microbiota supports the host plants with increased growth and production through various mechanisms such as P-solubilization, N-fixation, siderophore production, and rhizobacterial-induced drought endurance and resilience (RIDER) [17, 18]. Further, integrating microbes in the plantain breeding programs may benefit from varietal attributes which serve as an important tool for evaluating the production constraints and farmer preference criteria [19]. Thus, the development of a sustainable approach for improving soil quality, identifying and enhancing abundance of beneficial microbial community, and disease suppression is key towards improved plantain production.

Several studies have demonstrated the ecological requirements for *Musa* spp. and technological approaches that could increase yields and production [20, 21]. However, very few studies have focused on indigenous knowledge of plantain production, constraints, variations, and biodiversity specifically microbial diversity across agroecological zones. Thus, a more effective and highly robust system targeting long-term food security and the economic concerns of the smallholder farmers in SSA needs to be identified. Considering the lack of knowledge on plantain-microbe associations and the requirement for a holistic approach to increase productivity, this preliminary study aimed to explore microbiomes in plantain-based production systems in SSA. The study focused on examining self-supporting microbial ecosystems and distribution in agroecologies that fall under two different agroecologies (HR, high rainfall forests and SV, derived savannas) in West and Central Africa (WCA). In addition, we examined the compositions and assemblages of microbiomes in the rhizosphere. Our study is the first to describe an inventory of bacterial and fungal community associated with plantain-based production systems in humid tropics with different agroecologies and seasonal regimes of SSA. With this preliminary study of core microbiomes, we established a model for studying plantain-microbe interactions and their mechanisms that serves as a baseline for the future plant health and production studies.

## Methods

### Study Site and Rhizosphere Sample Collection

Plantain-growing fields in one West African country (Nigeria) and two Central African countries (Cameroon and Gabon) were targeted for sampling. Data coordinates for each sampling point is represented in Table S1. In each country, agroecological zones falling under two different seasonal regimes (HR, high rainfall forests, rainfall > 1500 mm; and SV, derived savannas, rainfall < 1500 mm) were identified, representing the major plantain-growing regions. Because of the presence of highly rich and diverse microbial diversity, we focused on the smallholder farming systems where fields are predominantly managed with local traditional technologies. Plants with identical physiological stages and random selections were sampled. Around the root zone, 15–30-cm depth was chosen for the collection of rhizosphere soil samples, and at the similar depth after removal of attached soil, root samples were collected. For metagenomics study, both rhizosphere soils and root samples were pooled for each agroecology (HR and SV) representing smallholder farmers’ plantain fields in WCA. Samples were immediately placed in polythene sampling bags and carried to the laboratory in an ice-cold box. Samples were further kept at 4 °C in the laboratory until processed. All procedures were carried out according to the institutional guidelines and regulations (International Institute of Tropical Agriculture). Prior to sampling, permissions were obtained from each farmer.

### Genomic DNA Extraction and Amplicon Generation

The total bacterial DNA was extracted from 250 mg of soil using Plant and Soil DNA Kit (Zymo Research Corp., Irvine, CA, USA) according to the protocol. The bacterial universal V3-V4 region of the 16S rRNA gene was amplified with primers 27F (5′-AGAGTTTGATCCTGGCTCAG-3′) and 1492R (5′-GGTTACCTTGTTACGACTT-3′). The fungal universal ITS1 region was amplified with primers ITS1-F (5′-TCCGTAGGTGAACCTGCGG-3′) and ITS4 (5′-TCCTCCGCTTATTGATATGC-3′). For DNA extraction, 1-g soil sample was added to 15-mL centrifuge tube with glass beads and centrifuged at ≥ 10,000 × g for 1 min. The supernatant was thereafter transferred to collection tube and centrifuged at 8000 × g for 1 min. A total volume of 800 μL of Genomic Lysis Buffer was added to the filtrate in the collection tube for DNA extraction. The pellet obtained after centrifugation was used for further processing. Samples were homogenized by vortexing with an elution volume of 50 µL to ensure higher concentrations of DNA. Eluted DNA was then extracted in elution buffer and filtered. Total DNA concentration and purity was checked on 1% agarose gels.

### Illumina Sequencing Library Preparation

The paired-end libraries were generated using NEB Next Ultra DNA Library Prep Kit for Illumina (New England Biolabs) following the manufacturer’s recommendations with a 2 × 150 read length, and index codes were added. The library was quantified via Qubit and Q-PCR and Agilent Bioanalyzer 2100 system (Agilent Technologies). Finally, the libraries were sequenced on an Illumina MiSeq platform at BGI Pt Ltd., Beijing, China.

### Data Processing and Clustering

Paired-end reads were assigned to the same sample according to the unique barcodes and truncated by cutting off the barcode and primer sequences followed by merging using FLASH tool with a minimum 10-bp overlap to recreate the V3–V4 region. Quality filtering on the raw sequence reads was performed and the quality of clean sequence reads was detected by QIIME [22]. Chimeras were filtered out by using UCHIME (v4.2.40) [23]. The 16S rDNA and ITS sequences were screened for chimeras by mapping to the gold database (v20110519, UNITE (v20140703)) separately; de novo chimera detection was done for 18S rDNA sequences. After comparing with the reference database algorithm, chimera sequences were detected and removed to obtain the effective sequence reads.

### Analysis of Community Patterns

The sequence reads were clustered to operational taxonomic unit (OTU) by scripts of software USEARCH (v7.0.1090). The sequence reads were clustered into OTU with a 97% threshold by using UPARSE, and the OTU unique representative sequences were obtained. All sequence reads were mapped to each OTU representative sequences using USEARCH GLOBAL. Sequences with error > 1 and < 200 bp were removed followed by merging of forward and reverse reads for ITS1 sequences. OTU representative sequences were taxonomically classified using ribosomal database project (RDP) classifier v.2.2 trained on the Greengenes database, using 0.6 confidence values as cutoff [24].

### Databases Used for Species Annotation

16S rDNA is used for bacterial community: Greengene (default), V201305; RDP, Release9 201203 [25]. 18S rDNA is used for fungal community: Silva (default), V119 [26]. ITS is also used for fungal community: UNITE (default), Version6 20140910 [27]. Bioinformatics analysis pipeline for metagenomic analysis demonstrated in Figure S1.

### Data Mining

Alpha diversity was applied for analyzing the complexity of species diversity for a sample through several indices, including observed species, Chao1, ACE, and Shannon and Simpson indices. Observed species value, Chao1 value, and ACE value can reflect the species richness of the community, and the rarefaction curve was used to evaluate if produced data is enough to cover all species in the community. The indices were calculated by Mothur (v1.31.2), and the corresponding rarefaction curve were drawn by the package “Vegan” software R (v3.1.1). A Venn diagram could visually display the number of common/unique OTUs in multi-samples/groups. Based on OTU abundances, OTU of each group were listed, Venn diagram was drawn by Venn Diagram of software R (v3.1.1), then the common and specific OTU ID were summarized. Beta diversity analysis was used to evaluate differences of samples in species complexity by software QIIME (v1.80). To display the differences of OTU composition in different samples, principal coordinate analysis (PCoA) was used to construct 3D graph to summarize factors mainly responsible for this difference. Based on the OTU abundance information, the relative abundance of each OTU in each sample was calculated, and the PCoA of OTU was done with the relative abundance value by the package “ade4” of software R (v3.1.1). Unweighted pair group method with arithmetic mean (UPGMA) is a hierarchical clustering method using average linkage and was used to interpret the distance matrix produced by beta diversity. To measure the robustness of this result to sequencing efforts, we performed a jackknifing analysis, wherein 75% of the smallest sample sequences from each sample were chosen at random, and the resulting UPGMA tree from this subset of data was compared with the tree representing the entire available data set by QIIME. Anosim (analysis of similarities) was performed to reflect the similarities and differences of the community compositions and structure between groups and groups of different samples genus levels by color change.

## Results

### Physico-chemical Parameters of Study Sites

Physiological parameters of the three countries representing different sampling locations are given in Figure S2. Soil texture at sampling locations in Nigeria was from sandy clay to sandy loam, Gabon was clay loam, and Cameroon was from sandy clay to clay loam. Average soil pH ranging in samples from Nigeria was 5.9–7.1, Gabon was 6.5–7.4, and Cameroon was 5.8–7.3.

**Fig. 1:**
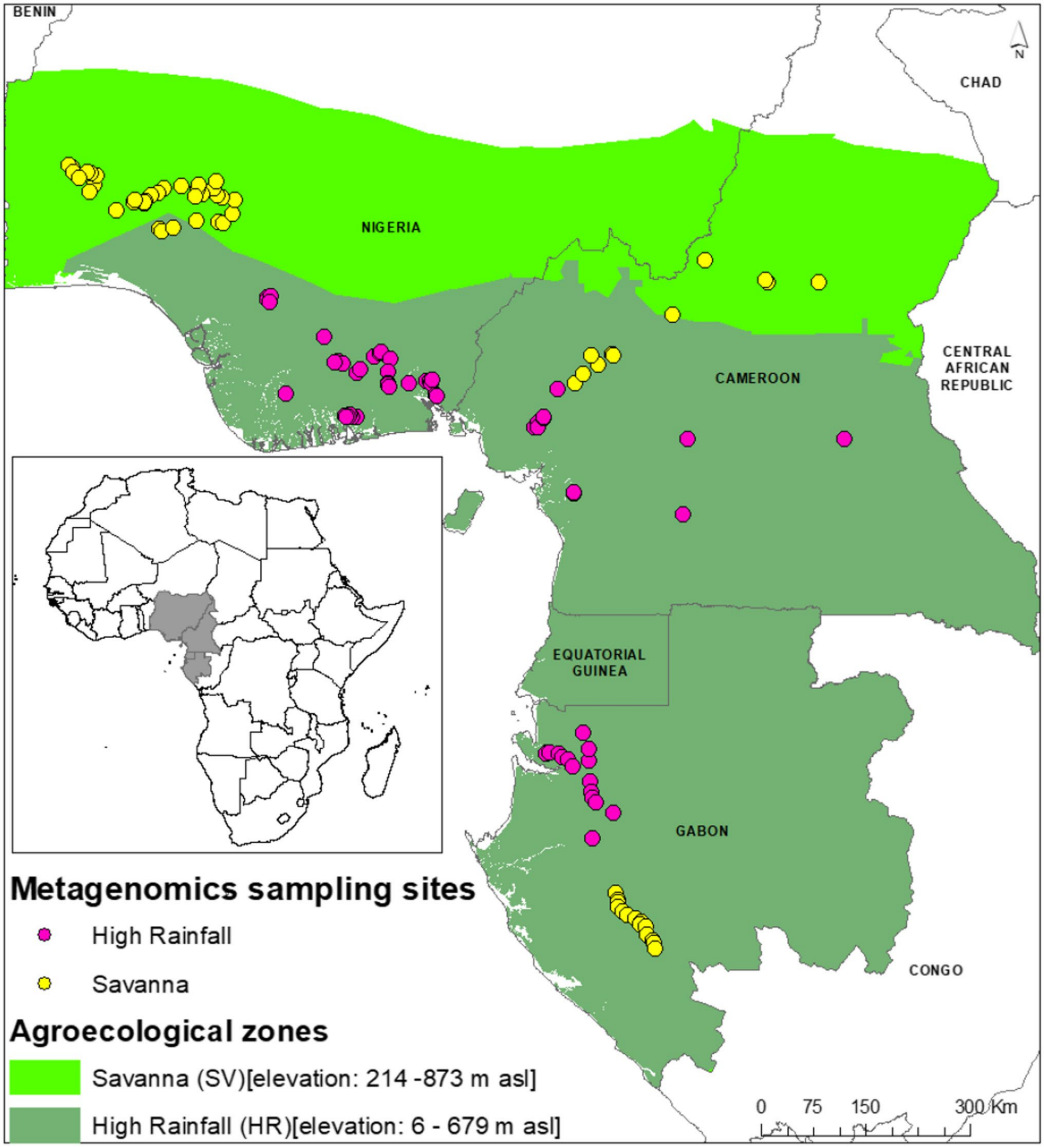
Area of study in West and Central Africa. Pink and yellow dots represent sampled plantain fields in high rainfall forests (HR) and derived savannas (SV), respectively

A total number of 286 samples were collected from fields (NHR, 96; NSV, 70; GHR, 28; GSV, 32; CHR, 30; CSV, 30) and pooled representing three countries (N, Nigeria; G, Gabon; and C, Cameroon), and two agroecologies (high rainfall forests (HR) and derived savannas (SV)). Details on the sampling points are given in the map (Fig. 1) and supplementary file (Table S1). This study was conducted to reveal the microbial diversity and differences in rhizosphere microbial communities between two plantain-growing agroecologies (HR and SV) in WCA (Fig. 1). The number of clean reads and mapped reads for 16S and ITS of each sample is summarized in Supplementary Tables S2 and S3. The tags, both in terms of bacteria and fungi, were clustered into OTUs at 97% shared sequence similarity.

### α-Diversity and Species Richness of Microbial Communities

To directly compare the α-diversity of samples, data was rarefied using QIIME and a rarefaction curve using Shannon diversity index was drawn that showed the richness of observed OTUs indicating that the sequencing data was sufficient to fully cover the whole diversity present in the samples (Figs. S3 and S4). In addition, the Chao1 estimator and accumulation curve of the OTUs richness yielded similar results suggesting that the sampling number was sufficient for detectable OTUs in each group (Figs. S3 and S4). The α-diversity of microbiome in SV was greater compared with that from HR (observed species (Sobs), Chao, ACE; *P* < 0.05). After the compartmentalization into niche areas (rhizosphere soil and root zones), α-diversity values did not differ significantly among the three countries (Figs. S5 and S6).

### Comparison of the Number of OTUs Between Agroecologies (HR and SV)

High differences were observed in OTUs richness between agroecologies (HR and SV). Among the total 2736 bacterial OTUs in both agroecologies, 493 and 617 were found in HR and SV of rhizosphere soil, respectively. Out of the total 233 bacterial OTUs, 48 and 51 were found in HR and SV of plantain roots, respectively (Fig. S7).

Identical OTU richness was observed for fungal community compositions where both agroecologies (HR and SV) differ from each other. Among the total 1102 fungal OTUs, 889 and 96 were found in HR and SV of rhizosphere soil, respectively. Out of total 671 fungal OTUs, 231 and 238 were found in HR and SV of plantain roots, respectively (Fig. S7). These results suggested that changes between HR and SV occurred to the most abundant bacterial communities, and some specific fungal communities dynamically responded to the seasonal regimes in both the agroecologies (Table S4).

### β-Diversity Among Different Samples

**Fig. 2:**
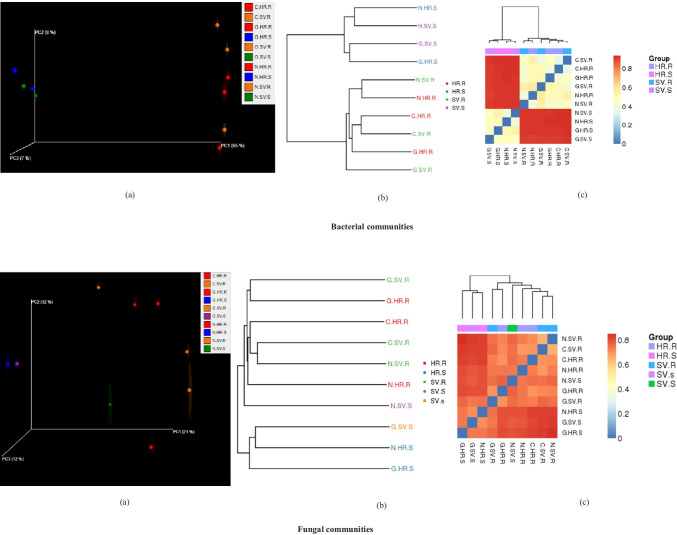
Similarities and differences in compositions and structures of bacterial and fungal communities among agroecologies and niche areas. **a** Principal coordinate analysis (PCoA) of pairwise, showing jackknife-supported confidence ellipsoids. The first three principal axes are shown. PCoA based on Euclidean distances. **b** Phylogenetic trees depicting patterns of relationships among agroecologies and niche areas. **c** β-Diversity heatmap showing sample clustering results among agroecologies and niche areas, where, C, Cameroon; G, Gabon; N, Nigeria; HR, high rainfall forests; SV, derived Savannas; S, rhizosphere soil; R, plantain roots

Principal coordinate analysis (PCoA) based on the differences in Bray–Curtis, unweighted and weighted UniFrac distances of microbial communities at the OTU level was performed to estimate β-diversity. A common pattern was observed using PCoA, with 65% and 21% differences in bacterial and fungal communities, respectively (Fig. 2A). Additionally, microbial populations at each sampling location were also significant (*P* < 0.05) between agroecologies. It was observed that the samples from the three countries were clustered together indicating similarities in the microbial communities occupying these premises (Fig. 2a). An evolutionary tree and heatmap at all the sampling locations was constructed using the UPGMA mean methods. No clear clades were observed among the three different countries (Nigeria, Cameroon, and Gabon) at the two agroecologies (Fig. 2b, c).

### Relative Abundance of Dominant Bacterial Taxonomy in Plantain Forelands

**Fig. 3:**
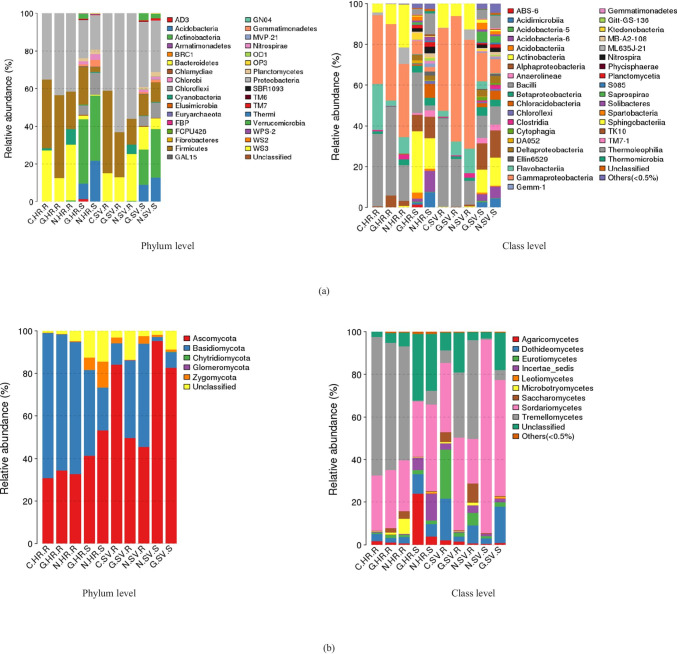
The composition and relative abundance of major bacterial and fungal taxa of the rhizosphere soil and plantain root-associated microbiome in HR and SV. **a** Bacterial communities, **b** fungal communities, where C, Cameroon; G, Gabon; N, Nigeria; HR, high rainfall forests; SV, derived Savannas; S, rhizosphere soil; R, plantain roots

Most phylotypes of bacterial populations in rhizosphere soil and plantain roots were detected in both HR and SV agroecologies, with only a few sticked to one regime and a single niche area (Fig. 3, Table S5). The frequently detected taxa with > 1% relative abundance at different classification levels in any ecology and niche area were further analyzed.

#### Changes at the Phylum Level

Across all the samples, we detected seven most abundant phyla (relative abundance higher than 1% at the phylum level in both the agroecologies). These include Proteobacteria, Firmicutes, Bacteroidetes, Actinobacteria, Acidobacteria, Chloroflexi, and cyanobacteria accounting for 92.9–99.0% of all bacterial taxa in the rhizosphere soil and roots of plantain at the two agroecologies. Proteobacteria dominated the rest of the six phyla in different samples and became the most abundant phylum (Fig. 3, Table S5). Also, most sequences (85.9%) belonged to members of three phyla: Proteobacteria (43.7%), Firmicutes (24.7%), and Bacteroidetes (17.6%). Strikingly, irrespective of small differences between the two agroecologies, microbial communities appeared to be more niche area associated, whose relative abundances were higher in plantain roots (*P* < 0.01). On the other hand, a significant presence of Acidobacteria and Chloroflexi were only found in rhizosphere soil of both agroecologies.

#### Changes at the Lower Levels

A total of 38 classes were detected with 10 being abundant (relative abundance > 1% at both agroecologies). Some phyla had the same dominant classes. For example, in Proteobacteria, the classes Gammaproteobacteria and in Bacteroidetes, the classes Flavobacteriia dominated plantain roots; however, Thermoleophilia of the phylum Actinobacteria dominated in rhizosphere soil of HR and SV (Fig. 3). In the class Bacilli which is confined to both HR and SV, the order Bacillales dominated in rhizosphere soil and roots and accounted for 3.9–19.9%. The dominant order of classes Gammaproteobacteria was Pseudomonadales, which were equally present in all the roots samples of both agroecologies. However, Enterobacteriaceae of class Gammaproteobacteria was greatly confined to SV compared to HR agroecology.

**Fig. 4:**
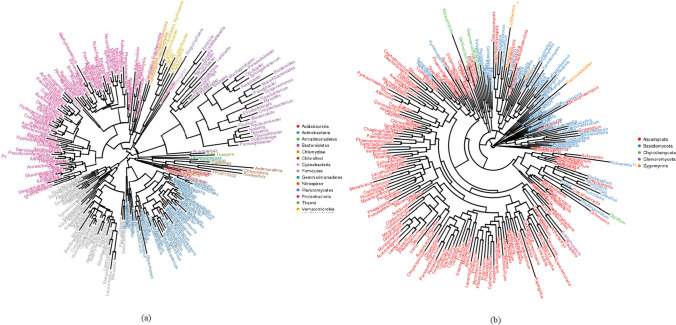
The genus species phylogeny tree displaying plantain associated microbiome in HR and SV. **a** Bacterial communities, **b** fungal communities

Phylogenetic trees were constructed for microbial genera from the rhizosphere. The bacteria identified in previous studies conducted in different countries obtained from NCBI were used to construct the phylogenetic trees (Fig. 4). In the neighbor-joining tree, 14 main phyla were observed for bacterial communities. In both the agroecologies, samples were highly associated with the microbes belonging to *Bacillus*, *Flavobacterium*, *Acinetobacter*, *Balneimonas*, *Pseudomonas*, and *Enterobacter*. Irrespective of agroecology, the dominant phyla in samples have the relative abundance of 94.3–97.8%. Apart, family Bacillaceae had a relatively much higher abundance in plantain roots of SV compared to HR ecology. In reverse, family Pseudomonadaceae had a relatively much higher abundance in plantain roots of HR compared to SV ecology. These results suggest that the overall microbial communities display alteration in HR and SV agroecologies.

### Relative Abundance of Dominant Fungal Taxonomy in Plantain Forelands

#### Changes at the Phylum Level

Ascomycota, Basidiomycota, and Zygomycota were the three most abundant fungal phyla detected across all samples in both agroecologies. Ascomycota dominated the rest of the five phyla with a relative abundance of 25.8–85.7% in different samples and became the most abundant phylum (Fig. 3, Table S6). Interestingly, fungal communities seemed to be more seasonal and agroecological associated, where we found higher relative abundances phylum—Ascomycota in SV compared to HR. In reverse, Basidiomycota dominated in HR compared to SV (Fig. 3, Table S6).

#### Changes at the Lower Levels

A total of 10 classes were detected with four being abundant (relative abundance > 1% at the class level at both agroecologies). Some phyla had the same dominant classes in each niche area. Ascomycota had the same classes Dothideomycetes (0.89–5.97%), Eurotiomycetes (1.8–9.8%), and Sordariomycetes (9.9–88.1%) dominated in both agroecologies (Fig. 3). Basidiomycota was dominated by Agaricomycetes which accounted only for 0.1–22.9% of the total taxa. Zygomycota was dominated by *Incertae sedis* which accounted for 4.8–11.9% of the total taxa. In the class Sordariomycetes, although the order Hypocreales dominated in both agroecologies accounted for 4.9–29.9%, a high relative abundance was found SV. Phylogenetic trees were constructed for microbial genera from the plantain rhizosphere. The fungi identified in previous studies conducted in different countries obtained from NCBI were used to construct the phylogenetic trees (Fig. 4). In the neighbor-joining tree, five main phyla were observed for fungal communities. Both the agroecologies were observed with very high dominance of *Trichosporon* spp. and *Fusarium* spp., whereas a high abundance of *Aspergillus* spp. (2.2%) were found in SV.

### Relationship Profiles in the Plantain Rhizosphere Microbes Between Agroecologies (HR and SV)

Hierarchically clustered heatmaps were generated to know the relationship among samples. Differences were observed among the samples and different agroecologies. These differences between microbial communities in HR and SV were also shown by clustering.

**Fig. 5:**
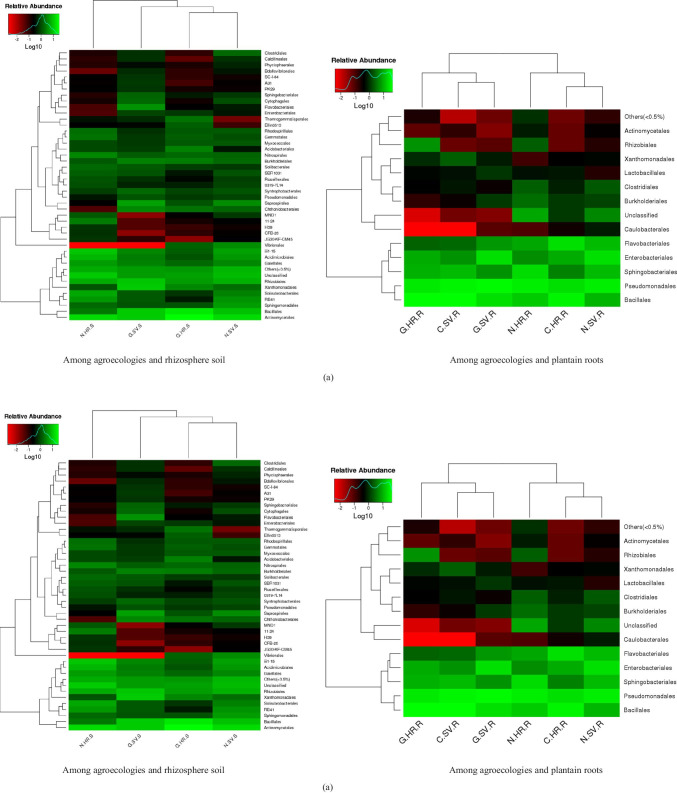
Hierarchically clustered heatmaps showing the relationship among samples in different agroecologies and niche areas. The closer the color to the dark represents the more dominant microbial community. **a** Bacterial communities, **b** fungal communities, where C, Cameroon; G, Gabon; N, Nigeria; HR, high rainfall forests; SV, derived Savannas; S, rhizosphere soil; R, plantain roots

According to the heatmaps, the fluctuation of the bacterial communities in SV was greater than HR (Fig. 5a). At the order level, C.SV.R and G.SV.R, C.HR.R and N.SV.R, and G.HR.S and N.SV.S clustered together, respectively. Clustering of HR and SV at family and order level showed that there were similar bacterial communities between HR and SV. In HR, the activity of bacteria was lower compared to SV, and there were very few dominant bacteria at the order and family level. The higher abundance of Sphingomonadales, Pseudomonadales, Bacillales, and Rhizobiales originating from both rhizosphere soil and plantain roots were observed.

According to heatmaps of fungal communities (Fig. 5b), the fungal communities in SV were more stable than HR. At the order level, the clustering of C.SV.R and N.SV.R assembled with the clustering of G.HR.S and N.HR.S. The G.HR.R and G.SV.R clustered together showing the similarity in the level of fungal communities. The higher abundance of *Incertae sedis*, Agaricales, and Trichosporonales were also observed.

### Analysis of Variations in the Functional Attributes of Plantain

To further investigate any potential difference in the functional diversity of the microbial communities among the agroecologies, functional annotations of the microbial community were obtained by blasting against the Clusters of Orthologous Genes (COGs) and Kyoto Encyclopedia of Genes and Genomes (KEGG) Orthology database. The obtained sequencing results showed no significant variations in the functionality of microbes among HR and SV.

**Fig. 6:**
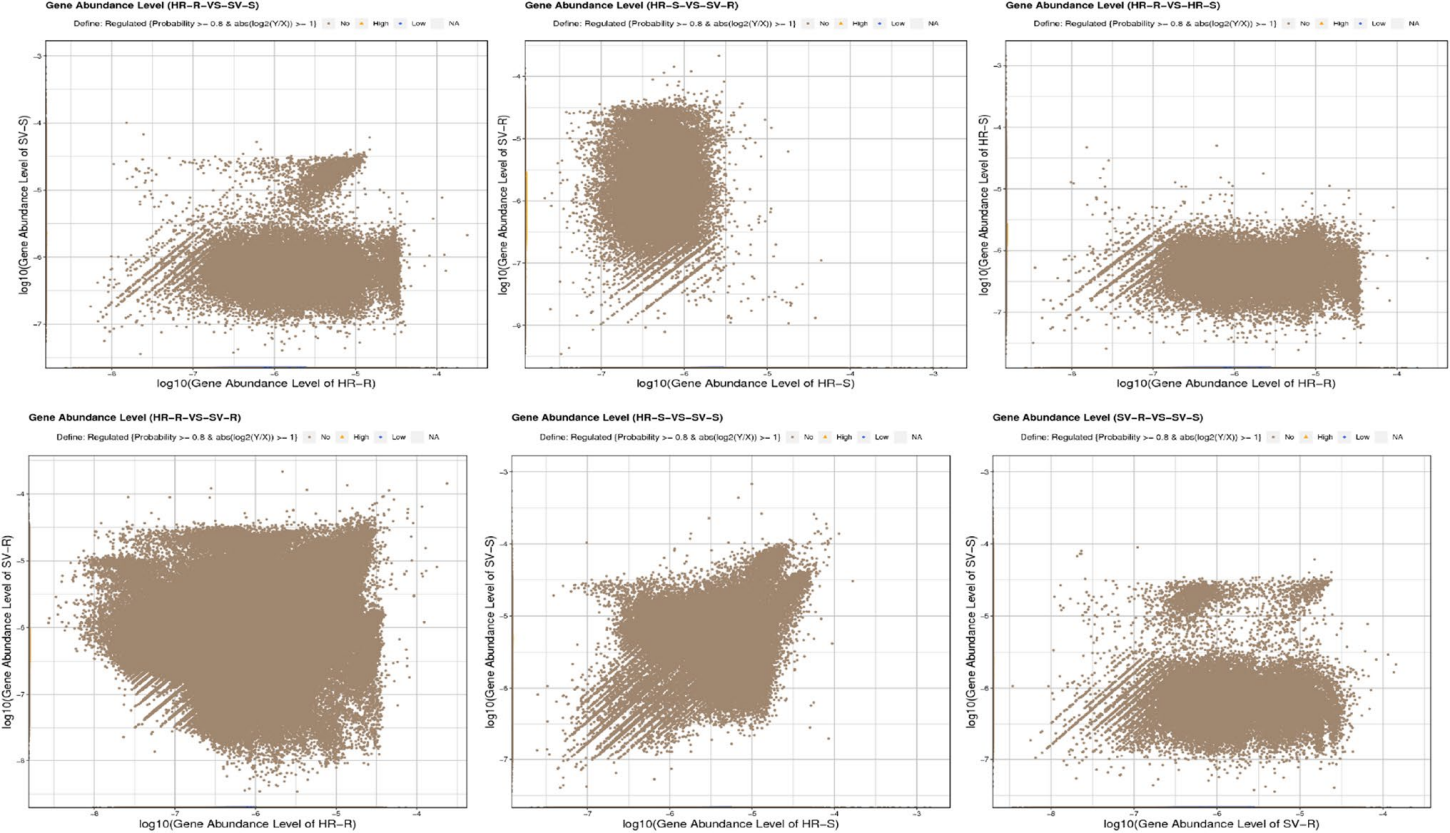
Scatter plot of gene expression levels in the compared groups of different agroecologies and niche areas, where C, Cameroon; G, Gabon; N, Nigeria; HR, high rainfall forests; SV, derived Savannas; S, rhizosphere soil; R, plantain roots

**Fig. 7:**
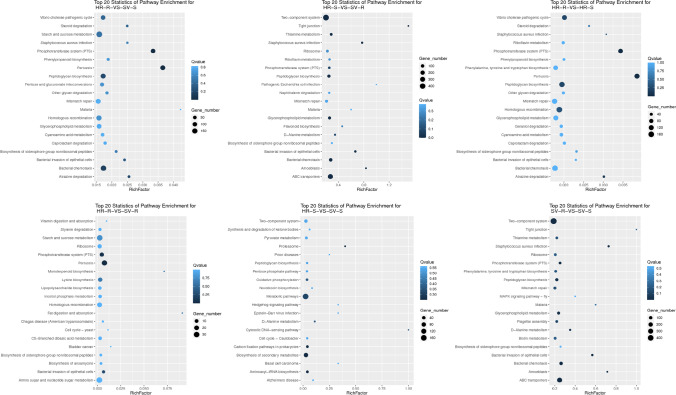
Pathway functional enrichment of the compared groups of different agroecologies and niche areas. X axis represents enrichment factor. Y axis represents pathway name. The color indicates the *q* value (high, white, low, blue); the lower *q* value indicates the more significant enrichment. Rich factor refers to the value of enrichment factor, which is the quotient of foreground value and background value (total gene amount). The larger the value, the more significant enrichment, where C, Cameroon; G, Gabon; N, Nigeria; HR, high rainfall forests; SV, derived Savannas; S, rhizosphere soil; R, plantain roots

KEGG enrichment analysis revealed the differentially abundant genes in the six comparison groups, i.e., HR-R vs. SV-S, HR-S vs. SV-R, HR-R vs. HR-S, HR-R vs. SV-R, HR-S vs. SV-S, and SV-R vs. SV-S. Among these groups, abundance of genes for HR-R vs. SV-S (high-16; low-18003), HR-S vs. SV-R (high-9523; low-813), HR-R vs. HR-S (high-668; low-20058), HR-R vs. SV-R (high-419; low-1880), HR-S vs. SV-S (high-51; low-727), and SV-R vs. SV-S (high-23; low-9244) were obtained. Except for HR-S vs. SV-R, there was an observed general trend toward differentially high and low abundant taxa. This was the most genetically distinct group (HR-S vs. SV-R) that had the greatest number of differentially highly abundant taxa (Fig. 6). A total of 20 KEGG pathways were identified in the six comparison groups which showed significant enrichment in all the compared groups of the HR and SV. The pathways with the greatest enrichment between HR-R vs. SV-S were bacterial chemotaxis, peptidoglycan biosynthesis, and phosphotransferase system (PTS). The HR-S vs. SV-R demonstrated the greatest enrichment of two component systems, ABC transporters, bacterial chemotaxis, glycerophospholipid metabolism, and peptidoglycan biosynthesis which corresponds to SV-R vs. SV-S. Enrichment of PTS and peptidoglycan biosynthesis were found in HR-R vs. HR-S. The HR-S vs. SV-S were enriched with metabolic pathways, biosynthesis of secondary metabolites, and carbon fixation pathways (Fig. 7).

## Discussion

To improve plant health and increase crop productivity and economic benefits to the smallholder farming community in SSA, one will require research in several directions. Harnessing the potential plant–microbe interactions should be at the forefront of such efforts. Like other crops, the merits of cultivating host-microbe interactions in bananas and plantains are two-fold, i.e., increasing productivity, and reducing the pressure of chemical fertilizers and pesticides on the environment that are making a huge economic impact globally [28]. Previous studies have reported that investigating specific regions of the 16S rDNA and ITS can elucidate the diversity profile when amplified for microbial community characterization [29]. Studies also revealed the efficacy of primers on microbial diversity profiles with the greatest relative abundances of the phyla and domains recovered from rhizosphere soils [30, 31]. In the current study, we adopted the amplification of V3–V4 region of the 16S rDNA and ITS in all the sample sets to enhance the production efficiency for enriched classifiable sequences. During morphological changes, perennial plants also observe root exudate variations, which results in the successional shift of microbial community in roots and close vicinity, i.e., rhizosphere soil of the plantains. This suggests that the plant species recruits specific microbiota regarding their functional attributes [32]. Therefore, we aimed to analyze the below-ground microbial communities targeting different agroecologies that fall under two different seasonal regimes (HR and SV) in three countries (Nigeria, Cameroon and Gabon) of WCA.

Microbial diversity was analyzed by α- and β-diversity indices. With regard to agroecologies, the α-diversity of the microbiome in SV was greater compared with that from HR (Figs. S5 and S6). According to PCoA, microbial communities from all sampling sites exhibited significant agroecological changes (Fig. 2). This change could be adapted due to the proximate connection and variations in root exudate’s profile of plantain rhizosphere. Microbial community enrichment in rhizosphere is a well-established and already known fact; however, our findings supplemented this fact by validating the concept that microbiome structure and composition alters with respect to diversified agroecologies (HR and SV). Root exudate metabolites are another primary driver of below-ground microbial communities [33, 34].

We further analyzed the microbial communities associated with plantain in two agroecologies (HR and SV) and observed a few taxa consistently enriched in the rhizosphere. Proteobacteria was obtained as the dominant phylum accounting for 24.9–57.5% of the total relative abundance. Endless bacterial communities in the Proteobacteria are particle-associated and play decisive roles in nitrogen cycling [35]. In the Proteobacteria, Bacillales and Pseudomonadales orders dominated in the rhizosphere of HR and SV (Fig. 3). In earlier studies, enrichment of proteobacteria was observed in the rhizosphere of various *Musa* spp. in Africa [36, 37]. A more stable relative abundance of Alphaproteobacteria in HR and SV agroecologies can be supported by previous studies which indicated that seasonal changes did not affect Alphaproteobacteria [38]. Firmicutes were the second abundant phylum in the plantain rhizosphere, consistent with the findings that they have been recovered from diverse habitats, such as soils, rivers, as symbionts within animals and plants [39] which could be due to their highly diverse phenotypic and metabolic activities. Actinobacteria was another dominant phylum with Actinomycetales as a consistently enriched taxon in the plantain rhizosphere with a relative abundance of 7.9–15.9% among both the agroecologies. Throughout history, Actinobacteria were considered the primary active group residents in rhizosphere [40]. The production of antimicrobial secondary metabolites by Actinomycetales serves as an elite choice selected due to host immune responses [41, 42].

Various alterations in root microbiome were observed in host plants at diversified environmental and developmental stages [43]. Consistent with such findings, our results demonstrated the changes in the microbial community profiles in different agroecologies and seasonal regimes. Niche areas’ analysis on relative abundances of the microbial communities revealed more rhizosphere dependent changes in the plantain microbiome. For example, among all the taxa studied at the genus level, *Bacillus, Flavobacterium, Acinetobacter*, *Balneimonas*, *Pseudomonas*, and *Enterobacter* were highly enriched in rhizosphere of plantains (Fig. 4). Enrichment of *Enterobacter* was often used as an indicator of the amendments in the plantain fields of both human and animal manure in these traditional smallholder farms. Besides the positive colonization of *Enterobacter* in the rhizosphere, it requires further research to analyze their pathogenicity in plantain.

Among fungal communities, Ascomycota, Basidiomycota, and Zygomycota were the three most abundant phyla observed in the rhizosphere plantains. In contrast with the bacterial communities and niche areas, fungal taxa were more observed under the influence of agroecologies (HR and SV) supporting the previous research findings indicating that the plant disease infection rates are highly influenced by seasonal changes [44]. Sordariomycetes is the only fungal class showing high diversity in each sample. Sordariomycetes are assumed to be cosmopolitan, with variability in functioning also including pathogens, endophytes of plants, and saprobes that are involved in decomposition. In our study, the majority of OTU sequences belonging to the Sordariomycetes and most likely play a role in the breakdown of organic material breakdown in a symbiotic or mutualistic relationship host. At the genus level, a very high relative abundance of *Trichosporon* spp. and *Fusarium* spp. were observed at both agroecologies (HR and SV). Further, SV showed increased enrichment of *Aspergillus* spp. in the rhizosphere of plantains (Fig. 4). These results suggest that the overall microbial communities display alteration in HR and SV agroecologies and the shifts in community composition between different sampling locations.

In any terrestrial ecosystem, along with microbial structure and compositions, studying the functional attributes is always needed because the changes in the diversity profile of rhizosphere are attributed to diversified functionalities possessed by the host plant microbiome. Therefore, to analyze the relationship between plantain host and bacterial diversity, functional attributes of the microbial communities in rhizosphere soil and plantain roots were studied by Clusters of Orthologous Genes (COG) and KEGG pathway’s functional classifications. KEGG enrichment analysis revealed the differentially abundant genes in the six comparison groups observed with a general trend toward differentially high and low abundant taxa except for HR-S vs. SV-R group (Fig. 6). In total, 20 KEGG pathways were found in significant enrichment in the six comparison groups of HR and SV. These most common pathways include bacterial chemotaxis, peptidoglycan biosynthesis, phosphotransferase system (PTS), two component systems, ABC transporters, metabolic pathways, biosynthesis of secondary metabolites, and carbon fixation pathways (Fig. 7). These pathways were associated with metabolism, signal transduction and defense, and transport, which involved the regulation of multiple plant–host interactions. The differences in the degree of enrichment and pathway specific enrichment suggest that responsive variability exist between the various rhizosphere soils and plantain roots responsible for structuring the microbial compositions in HR and SV. Variations in functional gene expressions between the different samples could be another reason for this enrichment [45, 46]. The composition of soil microbiome has always been recognized as a potent tool for any crop performance, its production and health in the field. For example, many pests and diseases such as banana weevil, nematodes, black Sigatoka disease, *Fusarium* wilt, and *Xanthomonas* wilt can suppress plant growth, while the build-up of efficient colonizers and beneficial microbes such as phosphate solubilizers and nitrogen fixers can enhance plant performance [47–49].

Finally, our approach of studying core microbiome and relative abundance through Bray–Curtis distance and α-diversity shows that plantain accommodates very specific associations in the rhizosphere. The potentially positive associations are the key to select and manipulate the microbiomes for plantain breeding with a competitive advantage. Nevertheless, manipulation of soil microbiota could also play a key role in suppressing pathogenic microorganisms and thus helps in improving the natural soil suppressiveness. This goal could be enhanced with the presence of highly beneficial microbiota that are necessary for plant health and production as revealed by our study. Our study also showed that plantains are a propitious habitat for microbial communities of beneficial bacteria and fungi specifically ideal for resource poor farming systems of Western and Central Africa.

## Conclusions

This study focused on the structure and composition of microbial communities with plantain rhizosphere under two different agroecologies and seasonal regimes in SSA. Metagenomics sequencing revealed higher diversities in both HR and SV and demonstrated obvious seasonal and agroecologies impact by complex shifts in both bacterial and fungal communities. In general, α-diversity of SV was greater than that from HR. In addition, relative abundances of the microbial communities revealed that some bacterial and fungal species varied quantitatively between the two agroecologies. The fate of COG and KEGG pathways and beneficial microbiome inhabiting plantain rhizosphere was also observed which further pointed out the need to enrich the current data with a functional analysis (such as metatranscriptomic and expressional studies) to better understand and profile the rhizosphere interactions between two agroecological variations. These studies further help in quantifying the beneficial effects of microbiota on the growth and production and address the success or failure of a defense strategies for controlling pathogenic microbes of plantain under small holder farming systems.

## Supplementary Information

Below is the link to the electronic supplementary material.

Supplementary file1 (XLSX 36 KB)

Supplementary file2 (DOCX 1193 KB)

## Data Availability

Sequences are accessible at the National Center for Biotechnology Information Sequence Read Archive under the accession PRJNA647667.

## References

[1] FAOSTAT (2020) Food and Agricultural Organisation of the United Nations. FAOSTAT statistical database. http://www.fao.org/faostat/en/#data/QC (April 2020)

[2] Ambachew ZG (2019). Review on impact of banana bacterial wilt (*Xanthomonas campestris* pv *musacerum*) in East and Central Africa. Cogent Food Agric.

[3] Selvaraj MG, Vergara A, Ruiz H, Safrai N, Elayabalan S, Ocimati W, Blomme G (2019). AI-powered banana diseases and pest detection. Plant Methods.

[4] Dita M, Barquero M, Heck D, Mizubuti ESG, Staver CP (2018). *Fusarium* wilt of banana: current knowledge on epidemiology and research needs toward sustainable disease management. Front Plant Sci.

[5] Bebber DP (2019). Climate change effects on Black Sigatoka disease of banana. Phil Trans R Soc.

[6] Hakim S, Naqqash T, Nawaz MS, Laraib I, Siddique MJ, Zia R, Mirza MS, Imran A (2021). Rhizosphere engineering with plant growth-promoting microorganisms for agriculture and ecological sustainability. Front Sustain Food Syst.

[7] Pande A, Mun BG, Lee DS, Khan M, Lee GM, Hussain A, Yun BW (2021). No network for plant–microbe communication underground: A review. Front Plant Sci.

[8] Lori M, Piton G, Symanczik S, Legay N, Brussaard L, Jaenicke S, Nascimento E, Reis F, Sousa JP, Mader P, Gattinger A, Clement JC, Foulquier A (2020). Compared to conventional, ecological intensive management promotes beneficial proteolytic soil microbial communities for agro-ecosystem functioning under climate change-induced rain regimes. Sci Rep.

[9] Tkacz A, Poole P (2021). The plant microbiome: The dark and dirty secrets of plant growth. Plants People Planet.

[10] Yin C, Casa Vargas JM, Schlatter DC, Hagerty CH, Hulbert SH, Paulitz TC (2021). Rhizosphere community selection reveals bacteria associated with reduced root disease. Microbiome.

[11] Kaushal M, Kumar A, Kaushal R (2017). *Bacillus pumilus* strain YSPMK11 as plant growth promoter and biocontrol agent against *Sclerotinia sclerotiorum*. 3 Biotech.

[12] Legein M, Smets W, Vandenheuvel D, Eilers T, Muyshondt B, Prinsen E, Samson R, Lebeer S (2020). Modes of action of microbial biocontrol in the phyllosphere. Front Microbiol.

[13] Tong AZ, Liu W, Liu Q, Xia GQ, Zhu JY (2021). Diversity and composition of the Panax ginseng rhizosphere microbiome in various cultivation modes and ages. BMC Microbiol.

[14] Trap J, Bonkowski M, Plassard C, Villenave C, Blanchart E (2016). Ecological importance of soil bacterivores for ecosystem functions. Plant Soil.

[15] Wang XY, Ge Y, Wang J (2017). Positive effects of plant diversity on soil microbial biomass and activity are associated with more root biomass production. J Plant Inter.

[16] Deng X, Zhang N, Shen Z, Zhu C, Liu H, Xu Z, Li R, Shen Q, Salles JF (2021). Soil microbiome manipulation triggers direct and possible indirect suppression against *Ralstonia solanacearum* and *Fusarium oxysporum*. NPJ Biofilms Microbiomes.

[17] Kaushal M, Wani SP (2016). Plant-growth-promoting rhizobacteria: drought stress alleviators to ameliorate crop production in drylands. Annal Microbiol.

[18] Kaushal M, Wani SP (2016). Rhizobacterial-plant interactions: strategies ensuring plant growth promotion under drought and salinity stress. Agric Ecosyst Environ.

[19] Ortiz R, Swennen R (2014). From crossbreeding to biotechnology-facilitated improvement of banana and plantain. Biotechnol Adv.

[20] Tenkouano A, Lamien N, Agogbua J, Amah D, Swennen R, Traore S, Thiemele D, Aby N, Kobenan K, Gnonhouri G, Yao N, Astin G, Sawadogo-Kabore S, Tarpaga V, Issa W, Lokossou B, Adjanohoun A, Amadji GL, Adangnitode S, Igue KAD, Ortiz R (2019). Promising high-yielding tetraploid plantain-bred hybrids in West Africa. Inter J Agron.

[21] Zorrilla-Fontanesi Y, Pauwels L, Panis B, Signorelli S, Vanderschuren H, Swennen R (2020). Strategies to revise agrosystems and breeding to control *Fusarium* wilt of banana. Nat Food.

[22] Gregory JC, Justin K, Jesse S (2010). QIIME allows analysis of high-throughput community sequencing data. Nat Methods.

[23] Edgar RC, Haas BJ, Clemente JC, Quince C, Knight R (2011). UCHIME improves sensitivity and speed of chimera detection. Bioinformatics.

[24] Cole JR, Wang Q, Fish JA, Chai B, McGarrell DM, Sun Y, Brown CT, Porras-Alfaro A, Kuske CR, Tiedje JM (2014). Ribosomal database project: data and tools for high throughput rRNA analysis. Nucleic Acids Res.

[25] DeSantis TZ, Hugenholtz P, Larsen N, Rojas M, Brodie EL, Keller K, Huber T, Dalevi D, Hu P, Andersen GL (2006). Greengenes, a Chimera-checked 16S rRNA gene database and workbench compatible with ARB. Appl Environ Microbiol.

[26] Quast C, Pruesse E, Yilmaz P, Gerken J, Schweer T, Yarza P, Peplies J, Glockner FO (2013). The SILVA ribosomal RNA gene database project: improved data processing and web-based tools. Nucleic Acids Res.

[27] Abarenkov K, Nilsson RH, Karl-Henrik L, Alexander IJ, Eberhardt U, Erland S, Hoiland K, Kjoller R, Larsson E, Pennanen T, Sen R, Taylor AFS, Tedersoo L, Ursing BM, Vralstad T, Liimatainen K, Peintner U, Koljalg U (2010). The UNITE database for molecular identification of fungi- recent updates and future perspectives. New Phytol.

[28] Deng L, Chen L, Zhao J, Wang R (2021). Comparative analysis on environmental and economic performance of agricultural cooperatives and smallholder farmers: the case of grape production in Hebei, China. PLoS ONE.

[29] Zhou J, Yu L, Zhang J, Liu J, Zou X (2021). Dynamic characteristics and co-occurrence patterns of microbial community in tobacco leaves during the 24-month aging process. Ann Microbiol.

[30] Fierer N, Jackson RB (2006). The diversity and biogeography of soil bacterial communities. Proc Natl Acad Sci USA.

[31] Jones P, Garcia BJ, Furches A, Tuskan GA, Jacobson D (2019). Plant host-associated mechanisms for microbial selection. Front Plant Sci.

[32] Hassani MA, Durán P, Hacquard S (2018). Microbial interactions within the plant holobiont. Microbiome.

[33] Olanrewaju OS, Ayangbenro AS, Glick BR, Babalola OO (2019). Plant health: feedback effect of root exudates-rhizobiome interactions. Appl Microbiol Biotechnol.

[34] Vieira S, Sikorski J, Dietz S, Herz K, Schrumpf M, Bruelheide H, Scheel D, Friedrich MW, Overmann J (2020). Drivers of the composition of active rhizosphere bacterial communities in temperate grasslands. ISME J.

[35] Ren M, Zhang Z, Wang X, Zhou Z, Chen D, Zeng H, Zhao S, Chen L, Hu Y, Zhang C, Liang Y, She Q, Zhang Y, Peng N (2018). Diversity and contributions to nitrogen cycling and carbon fixation of soil salinity shaped microbial communities in Tarim Basin. Front Microbiol.

[36] Kaushal M, Mahuku G, Swennen R (2020). Metagenomic insights of the root colonizing microbiome associated with symptomatic and non-symptomatic bananas in *Fusarium* wilt infected fields. Plants.

[37] Kaushal M, Swennen R, Mahuku G (2020). Unlocking the microbiome communities of banana (*Musa* spp.) under disease stressed (*Fusarium* wilt) and non-stressed conditions. Microorganisms.

[38] Lazzaro A, Brankatschk R, Zeyer J (2012). Seasonal dynamics of nutrients and bacterial communities in unvegetated alpine glacier forefields. Appl Soil Ecol.

[39] Newton RJ, McMahon KD (2011). Seasonal differences in bacterial community composition following nutrient additions in a eutrophic lake. Environ Microbiol.

[40] Goodfellow M, Williams ST (1983). Ecology of actinomycetes. Annu Rev Microbiol.

[41] Firáková S, Šturdíková M, Múčková M (2007). Bioactive secondary metabolites produced by microorganisms associated with plants. Biologia.

[42] Behie SW, Bonet B, Zacharia VM, McClung DJ, Traxler MF (2017). Molecules to ecosystems: Actinomycete natural products *in situ*. Front Microbiol.

[43] Yuan J, Zhao J, Wen T, Zhao M, Li R, Goossens P, Huang Q, Bai Y, Vivanco JM, Kowalchuk GA, Berendsen RL, Shen Q (2018). Root exudates drive the soil-borne legacy of aboveground pathogen infection. Microbiome.

[44] Velásquez AC, Castroverde CDM, He SY (2018). Plant–pathogen warfare under changing climate conditions. Curr Biol.

[45] Paczkowska M, Barenboim J, Sintupisut N, Fox NS, Zhu H, Abd-Rabbo D, Mee WM, Boutros PC, Reimand J, PCAWG drivers and functional interpretation working group, PCAWG Consortium (2020). Integrative pathway enrichment analysis of multivariate omics data. Nat Commun.

[46] Kaushal M, Mahuku G, Swennen R (2021). Comparative transcriptome and expression profiling of resistant and susceptible banana cultivars during infection by *Fusarium oxysporum*. Int J Mol Sci.

[47] Compant S, Saikkonen K, Mitter B, Campisano A, Mercado-Blanco J (2016). Editorial special issue: soil, plants and endophytes. Plant Soil.

[48] Alori ET, Glick BR, Babalola OO (2017). Microbial phosphorus solubilization and its potential for use in sustainable agriculture. Front Microbiol.

[49] Bargaz A, Lyamlouli K, Chtouki M, Zeroual Y, Dhiba D (2018). Soil microbial resources for improving fertilizers efficiency in an integrated plant nutrient management system. Front Microbiol.

