# Supplementary material for: Subterranean Microbiome Affiliations of Plantain (Musa spp.) Under Diverse Agroecologies of Western and Central Africa
Source: Microb Ecol. 2021 Sep 29;84(2):580–93. doi: 10.1007/s00248-021-01873-x (PMC9436888; doi:10.1007/s00248-021-01873-x)
Supplement: Supplementary file 2 — Supplementary file2 (DOCX 1193 KB) [file 248_2021_1873_MOESM2_ESM.docx]

**Supplementary Tables**

**Table S2**Summary of Illumina Miseq reads for each sample (16S). Where, C: Cameroon; G: Gabon; N: Nigeria; HR: High rainfall; SV: Savannas; S: Rhizosphere soil; R: Plantain roots

| **Sample Name** | **Reads Length (bp)** | **Raw Data (Mbp)** | **Adapter (%)** | **Nbase (%)** | **PloyBase (%)** | **Low Quality (%)** | **Clean Data (Mbp)** | **Data Utilization Ratio (%)** | **Raw Reads** | **Clean Reads** | **Read Utilization Ratio (%)** |
| --- | --- | --- | --- | --- | --- | --- | --- | --- | --- | --- | --- |
| C.HR.R | 299:293 | 21.96 | 0 | 0.04 | 0 | 2.106 | 20.93 | 95.33 | 37087*2 | 35693*2 | 96.24 |
| C.SV.R | 297:298 | 21.98 | 0 | 0.032 | 0.003 | 1.796 | 20.98 | 98.25 | 37891*2 | 36125*2 | 97.39 |
| G.HR.R | 296:294 | 21.54 | 0 | 0.037 | 0.002 | 1.891 | 20.62 | 95.74 | 36504*2 | 35256*2 | 96.58 |
| G.HR.S | 298:298 | 21.66 | 0 | 0.081 | 0.003 | 2.33 | 20.54 | 94.81 | 36349*2 | 34838*2 | 95.84 |
| G.SV.R | 294:294 | 21.72 | 0 | 0.031 | 0.004 | 1.754 | 20.86 | 96.03 | 36943*2 | 35790*2 | 96.88 |
| G.SV.S | 297:294 | 21.98 | 0 | 0.026 | 0.005 | 2.242 | 20.93 | 95.21 | 37198*2 | 35748*2 | 96.1 |
| N.HR.R | 299:294 | 21.85 | 0 | 0.029 | 0.003 | 1.888 | 20.96 | 95.93 | 36847*2 | 35615*2 | 96.66 |
| N.HR.S | 294:295 | 21.84 | 0 | 0.04 | 0.013 | 2.285 | 20.7 | 94.77 | 37085*2 | 35539*2 | 95.83 |
| N.SV.R | 293:294 | 21.56 | 0 | 0.033 | 0.001 | 2.063 | 20.6 | 95.56 | 36726*2 | 35389*2 | 96.36 |
| N.SV.S | 299:298 | 21.66 | 0 | 0.058 | 0.009 | 2.276 | 20.56 | 94.92 | 36282*2 | 34815*2 | 95.96 |

**Table S3**Summary of Illumina Miseq reads for each sample (ITS). Where, C: Cameroon; G: Gabon; N: Nigeria; HR: High rainfall; SV: Savannas; S: Rhizosphere soil; R: Plantain roots

| **Sample Name** | **Reads Length (bp)** | **Raw Data (Mbp)** | **Adapter (%)** | **Nbase (%)** | **PloyBase (%)** | **Low Quality (%)** | **Clean Data (Mbp)** | **Data Utilization Ratio (%)** | **Raw Reads** | **Clean Reads** | **Read Utilization Ratio (%)** |
| --- | --- | --- | --- | --- | --- | --- | --- | --- | --- | --- | --- |
| C.HR.R | 250:250 | 40.35 | 0 | 0.077 | 0.099 | 0.335 | 40 | 99.12 | 80708*2 | 80026*2 | 99.15 |
| C.SV.R | 250:250 | 35.21 | 0 | 0.037 | 0.567 | 0.627 | 20.89 | 98.25 | 48235*2 | 47893*2 | 98.99 |
| G.HR.R | 250:250 | 40.66 | 0 | 0.035 | 0.565 | 0.608 | 40 | 98.37 | 81323*2 | 80029*2 | 98.41 |
| G.HR.S | 250:250 | 21.1 | 0 | 0.043 | 0.492 | 0.666 | 20.72 | 98.2 | 42199*2 | 41469*2 | 98.27 |
| G.SV.R | 250:250 | 21.68 | 0 | 0.034 | 2.266 | 0.87 | 20.87 | 96.28 | 43352*2 | 41764*2 | 96.34 |
| G.SV.S | 250:250 | 21.32 | 0 | 0.039 | 2.298 | 0.716 | 20.51 | 96.18 | 42650*2 | 41047*2 | 96.24 |
| N.HR.R | 250:250 | 35.58 | 0 | 0.073 | 0.596 | 0.529 | 35 | 98.38 | 71155*2 | 70038*2 | 98.43 |
| N.HR.S | 250:250 | 21.17 | 0 | 0.028 | 0.352 | 0.616 | 20.86 | 98.52 | 42346*2 | 41753*2 | 98.6 |
| N.SV.R | 250:250 | 30.54 | 0 | 0.041 | 0.732 | 0.593 | 30 | 98.22 | 61087*2 | 60029*2 | 98.27 |
| N.SV.S | 250:250 | 21.2 | 0 | 0.101 | 1.205 | 0.632 | 20.66 | 97.47 | 42392*2 | 41343*2 | 97.53 |

**Table S4**Analysis of similarities (ANOSIM) Where, C: Cameroon; G: Gabon; N: Nigeria; HR: High rainfall; SV: Savannas; S: Rhizosphere soil; R: Plantain roots

**16S ITS**

| Group Name (Factor Name) | p value | R value | Level |  |
| --- | --- | --- | --- | --- |
| HR.R_vs_SV.R | 0.8 | -0.222 | anosim/genus | |
| HR.S_vs_SV.S | 0.333333 | 0.75 | anosim/genus | |
| HR.R_vs_HR.S | 0.4 | 0 | anosim/genus | |
| SV.R_vs_SV.S | 0.5 | 0 | anosim/genus | |

| Group Name (Factor Name) | p value | R value | Level |  |
| --- | --- | --- | --- | --- |
| HR.R_vs_SV.R | 1 | -0.111 | anosim/genus | |
| HR.S_vs_SV.S | 0.333333 | 1 | anosim/genus | |
| HR.R_vs_HR.S | 0.8 | -0.25 | anosim/genus | |
| SV.R_vs_SV.S | 0.25 | 1 | anosim/genus | |

**Table S5**Major bacterial taxa in samples. Where, C: Cameroon; G: Gabon; N: Nigeria; HR: High rainfall; SV: Savannas; S: Rhizosphere soil; R: Plantain roots

| Taxon | C.HR.R | G.HR.R | N.HR.R | G.HR.S | N.HR.S | C.SV.R | G.SV.R | N.SV.R | G.SV.S | N.SV.S |
| --- | --- | --- | --- | --- | --- | --- | --- | --- | --- | --- |
| Archaea;Euryarchaeota | 0 | 0 | 0 | 6 | 0 | 0 | 0 | 0 | 0 | 0 |
| Bacteria;AD3 | 0 | 0 | 0 | 296 | 7 | 0 | 0 | 0 | 8 | 4 |
| Bacteria;Acidobacteria | 2 | 1 | 1 | 1911 | 4394 | 1 | 3 | 3 | 1516 | 2697 |
| Bacteria;Actinobacteria | 17 | 21 | 172 | 7910 | 7001 | 43 | 11 | 82 | 3168 | 5522 |
| Bacteria;Armatimonadetes | 0 | 0 | 0 | 0 | 0 | 0 | 0 | 0 | 4 | 11 |
| Bacteria;BRC1 | 0 | 0 | 0 | 0 | 5 | 0 | 0 | 0 | 3 | 7 |
| Bacteria;Bacteroidetes | 6341 | 3087 | 7146 | 462 | 128 | 3736 | 3276 | 5617 | 2073 | 1215 |
| Bacteria;Chlamydiae | 0 | 0 | 0 | 63 | 2 | 0 | 0 | 0 | 58 | 39 |
| Bacteria;Chlorobi | 0 | 0 | 0 | 10 | 14 | 0 | 1 | 0 | 12 | 11 |
| Bacteria;Chloroflexi | 2 | 3 | 0 | 1196 | 2360 | 1 | 3 | 1 | 915 | 1669 |
| Bacteria;Cyanobacteria | 276 | 19 | 2008 | 26 | 57 | 13 | 8 | 1120 | 15 | 93 |
| Bacteria;Elusimicrobia | 0 | 0 | 0 | 10 | 2 | 0 | 0 | 0 | 1 | 7 |
| Bacteria;FBP | 0 | 0 | 0 | 0 | 0 | 0 | 0 | 0 | 0 | 11 |
| Bacteria;FCPU426 | 0 | 0 | 0 | 11 | 0 | 0 | 0 | 0 | 0 | 1 |
| Bacteria;Fibrobacteres | 0 | 0 | 0 | 0 | 4 | 0 | 0 | 0 | 1 | 5 |
| Bacteria;Firmicutes | 8476 | 10962 | 4784 | 4665 | 520 | 11160 | 6175 | 3126 | 1938 | 2250 |
| Bacteria;GAL15 | 0 | 0 | 0 | 79 | 68 | 0 | 0 | 0 | 14 | 3 |
| Bacteria;GN04 | 0 | 0 | 0 | 2 | 0 | 0 | 0 | 0 | 0 | 0 |
| Bacteria;Gemmatimonadetes | 0 | 1 | 0 | 182 | 704 | 0 | 0 | 0 | 178 | 311 |
| Bacteria;MVP-21 | 0 | 0 | 0 | 0 | 0 | 0 | 0 | 0 | 0 | 8 |
| Bacteria;Nitrospirae | 0 | 0 | 1 | 390 | 633 | 0 | 1 | 0 | 243 | 345 |
| Bacteria;OD1 | 0 | 0 | 0 | 4 | 2 | 0 | 0 | 0 | 13 | 26 |
| Bacteria;OP3 | 0 | 0 | 0 | 0 | 0 | 0 | 0 | 0 | 0 | 3 |
| Bacteria;Other | 0 | 0 | 0 | 10 | 27 | 0 | 0 | 0 | 6 | 24 |
| Bacteria;Planctomycetes | 1 | 0 | 0 | 398 | 485 | 0 | 0 | 0 | 184 | 449 |
| Bacteria;Proteobacteria | 8217 | 10861 | 10048 | 4671 | 3695 | 10415 | 16196 | 12599 | 5875 | 5797 |
| Bacteria;SBR1093 | 0 | 0 | 0 | 0 | 0 | 0 | 0 | 0 | 0 | 4 |
| Bacteria;TM6 | 0 | 0 | 0 | 36 | 3 | 0 | 0 | 0 | 26 | 20 |
| Bacteria;TM7 | 0 | 1 | 7 | 75 | 44 | 0 | 0 | 0 | 114 | 242 |
| Bacteria;Thermi | 0 | 0 | 0 | 0 | 5 | 0 | 0 | 0 | 6 | 1 |
| Bacteria;Verrucomicrobia | 0 | 0 | 0 | 625 | 19 | 0 | 1 | 0 | 635 | 505 |
| Bacteria;WPS-2 | 0 | 0 | 0 | 36 | 76 | 0 | 0 | 0 | 0 | 9 |
| Bacteria;WS2 | 0 | 0 | 0 | 0 | 6 | 0 | 0 | 0 | 0 | 5 |
| Bacteria;WS3 | 0 | 0 | 0 | 33 | 48 | 0 | 0 | 0 | 3 | 46 |

**Table S6**Major fungal taxa in samples. Where, C: Cameroon; G: Gabon; N: Nigeria; HR: High rainfall; SV: Savannas; S: Rhizosphere soil; R: Plantain roots

| Taxon | C.HR.R | G.HR.R | N.HR.R | G.HR.S | N.HR.S | C.SV.R | G.SV.R | N.SV.R | N.SV.S | G.SV.S |
| --- | --- | --- | --- | --- | --- | --- | --- | --- | --- | --- |
| Fungi;Ascomycota | 23285 | 26207 | 20283 | 13979 | 18037 | 21678 | 17504 | 23589 | 36392 | 28496 |
| Fungi;Basidiomycota | 51839 | 48959 | 38731 | 13650 | 6824 | 2578 | 12697 | 25114 | 755 | 2570 |
| Fungi;Chytridiomycota | 0 | 29 | 4 | 10 | 16 | 0 | 5 | 19 | 5 | 35 |
| Fungi;Glomeromycota | 0 | 0 | 0 | 0 | 0 | 3 | 24 | 0 | 0 | 0 |
| Fungi;Unclassified | 0 | 0 | 0 | 28 | 44 | 59 | 0 | 0 | 114 | 209 |
| Fungi;Zygomycota | 198 | 127 | 206 | 1988 | 4180 | 690 | 90 | 1858 | 395 | 374 |

**Supplementary Figures**

**
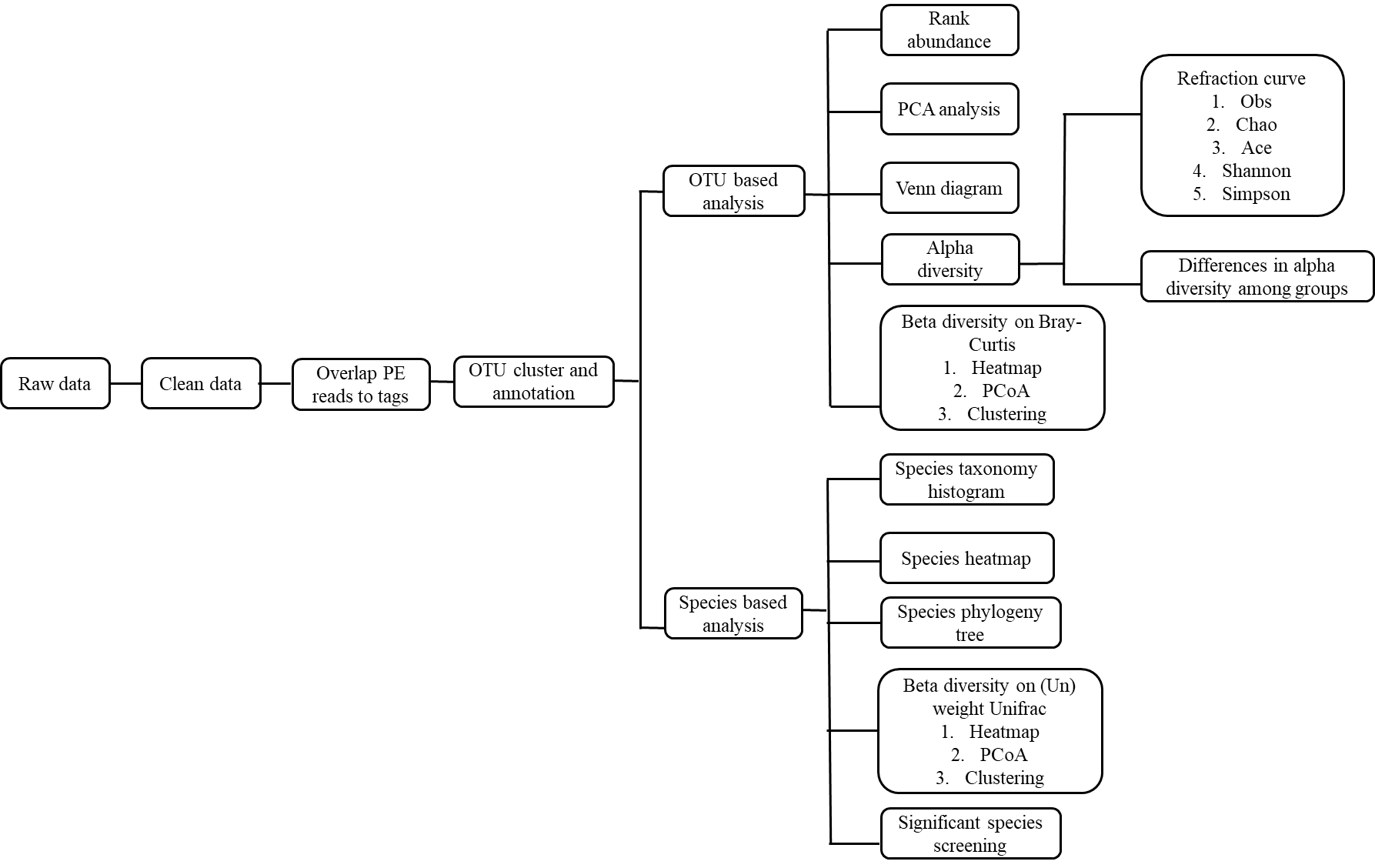
**

**Fig. S1**Bioinformatics analysis pipeline for meta-genomic analysis.

**Fig. S2**Physiological parameters of the countries representing different sampling locations.


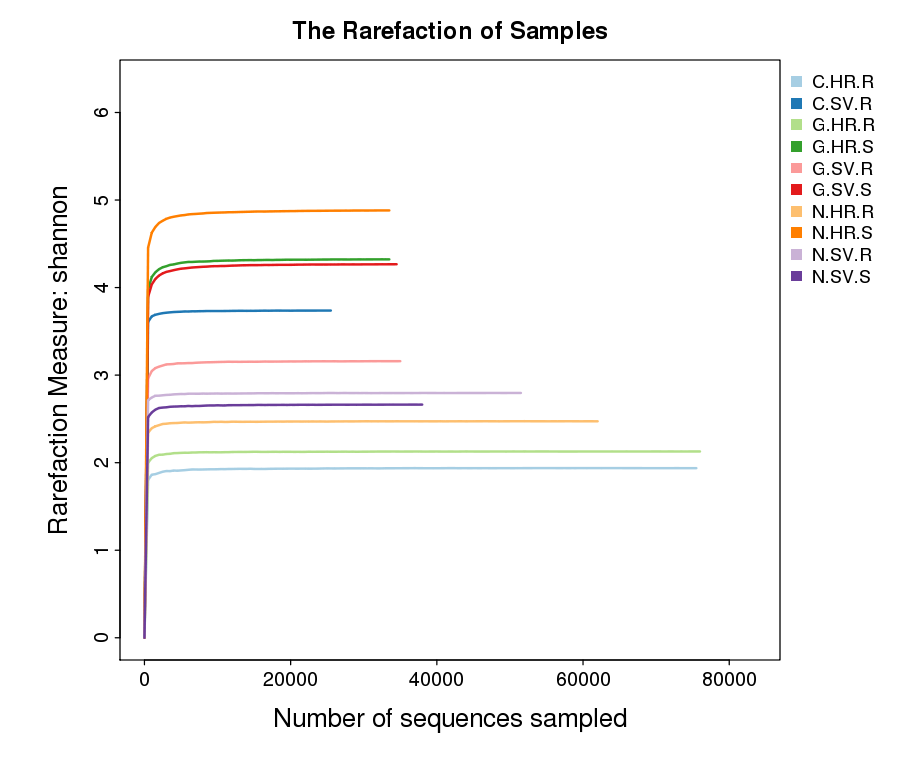

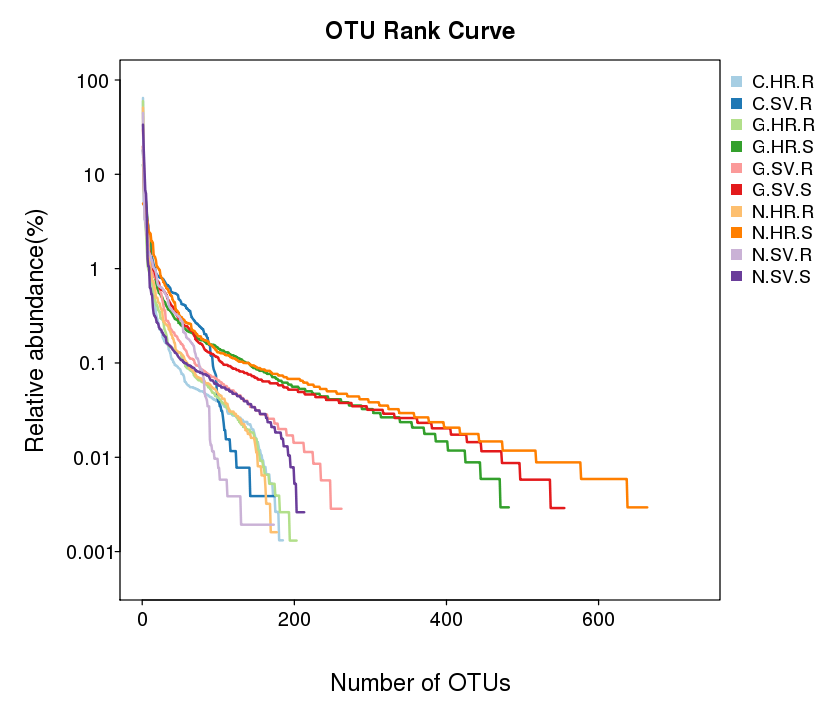


**Fig. S3**Alpha diversity of samples (16S). Where, C: Cameroon; G: Gabon; N: Nigeria; HR: High rainfall; SV: Savannas


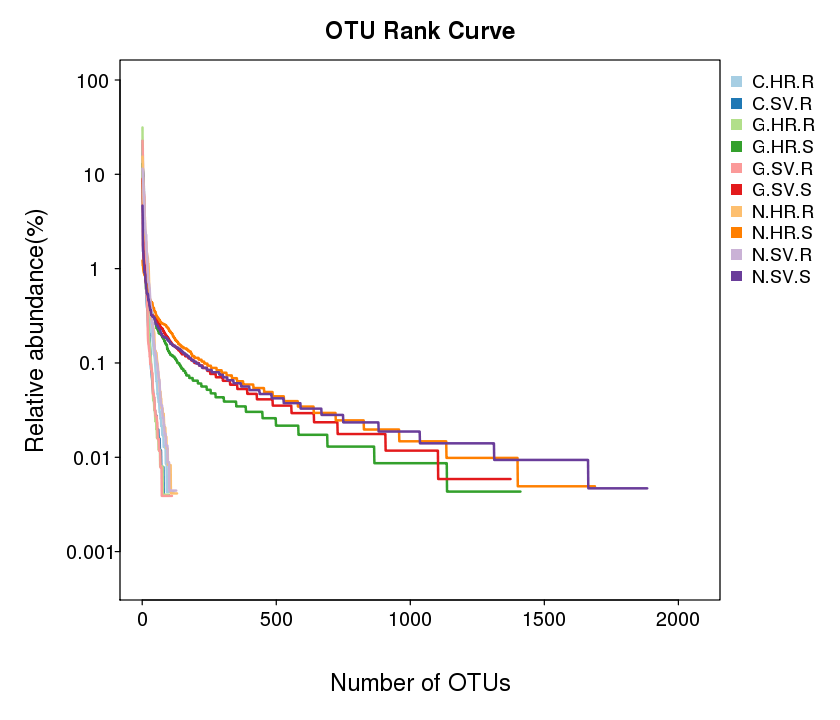

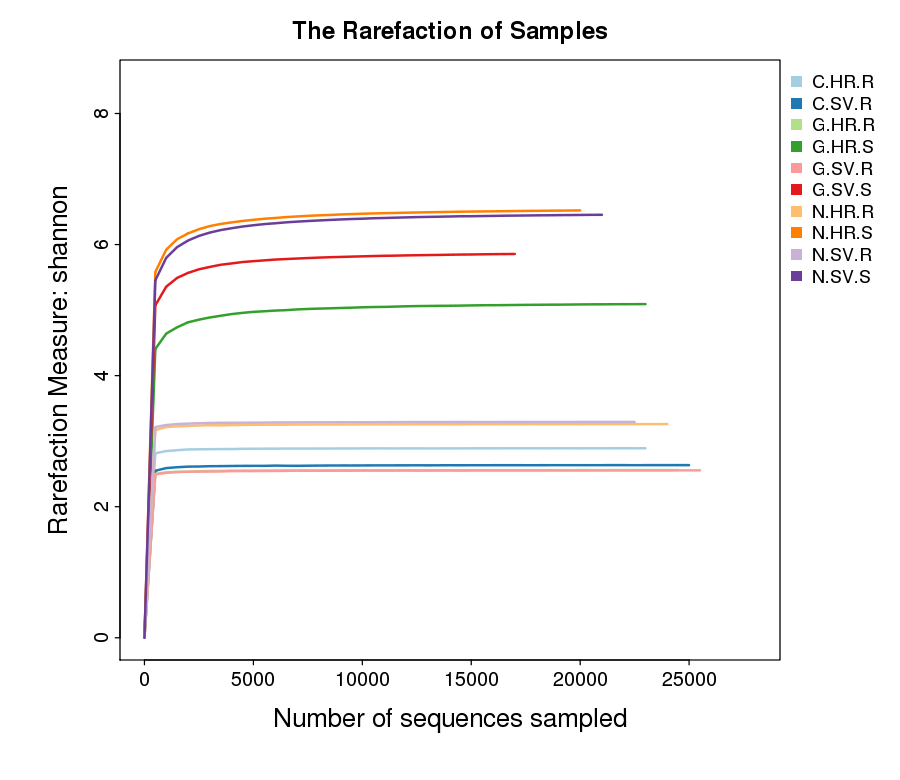


**Fig. S4**Alpha diversity of samples (ITS). Where, C: Cameroon; G: Gabon; N: Nigeria; HR: High rainfall; SV: Savannas


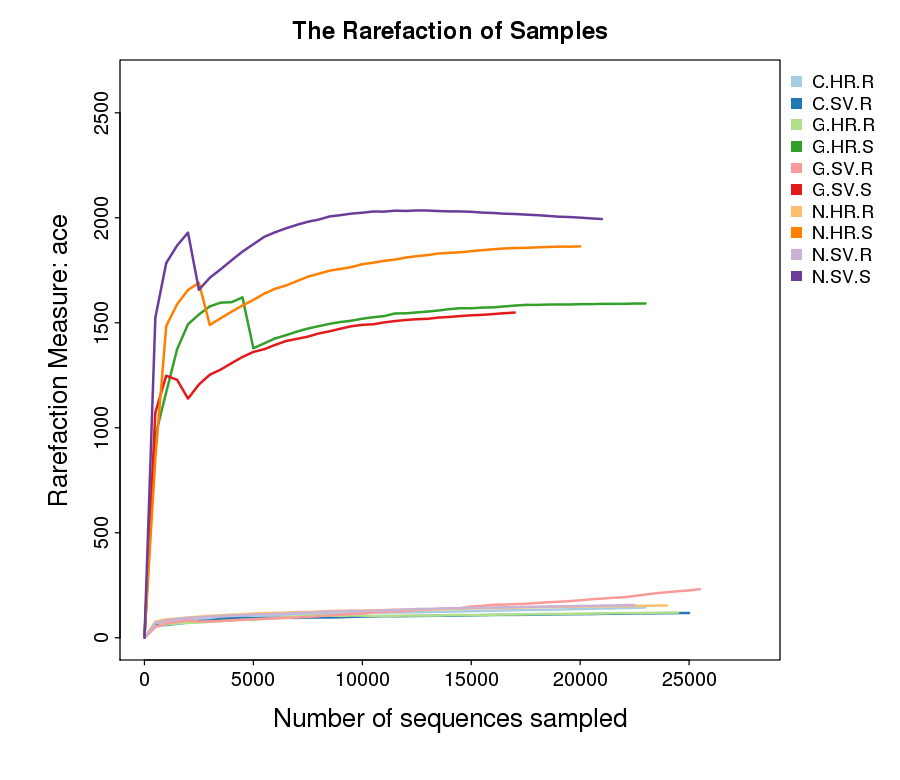

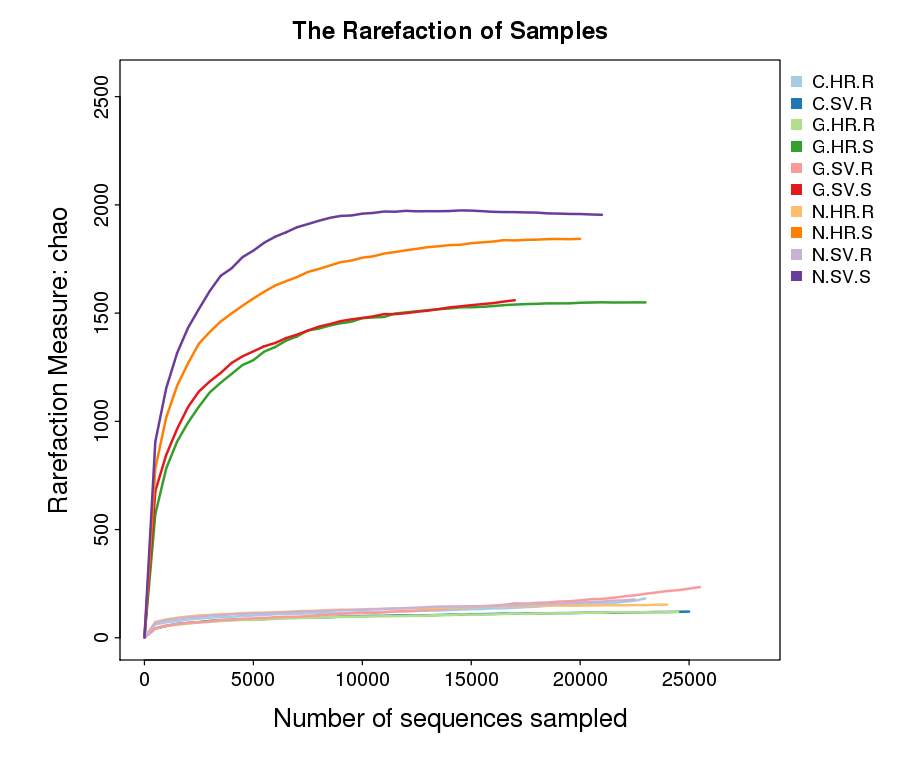


A B

**Fig. S5**Alpha diversity of bacterial communities indicating the biodiversity of the samples. A. Observed species, ACE; P < 0.05 B. Observed species, Chao; P < 0.05. Where, C: Cameroon; G: Gabon; N: Nigeria; HR: High rainfall; SV: Savannas


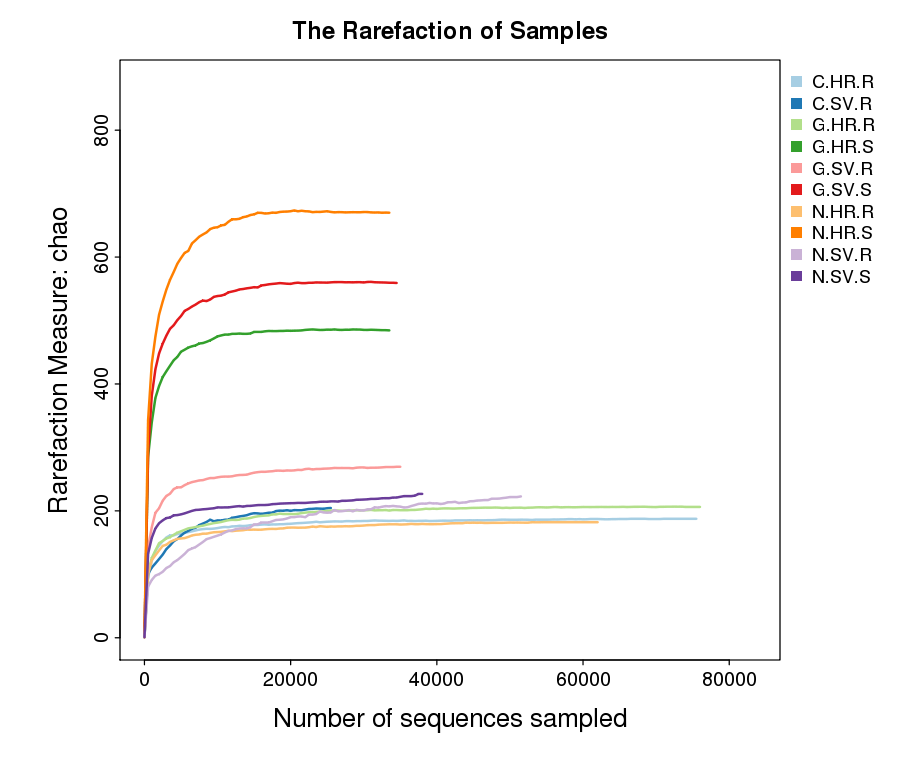

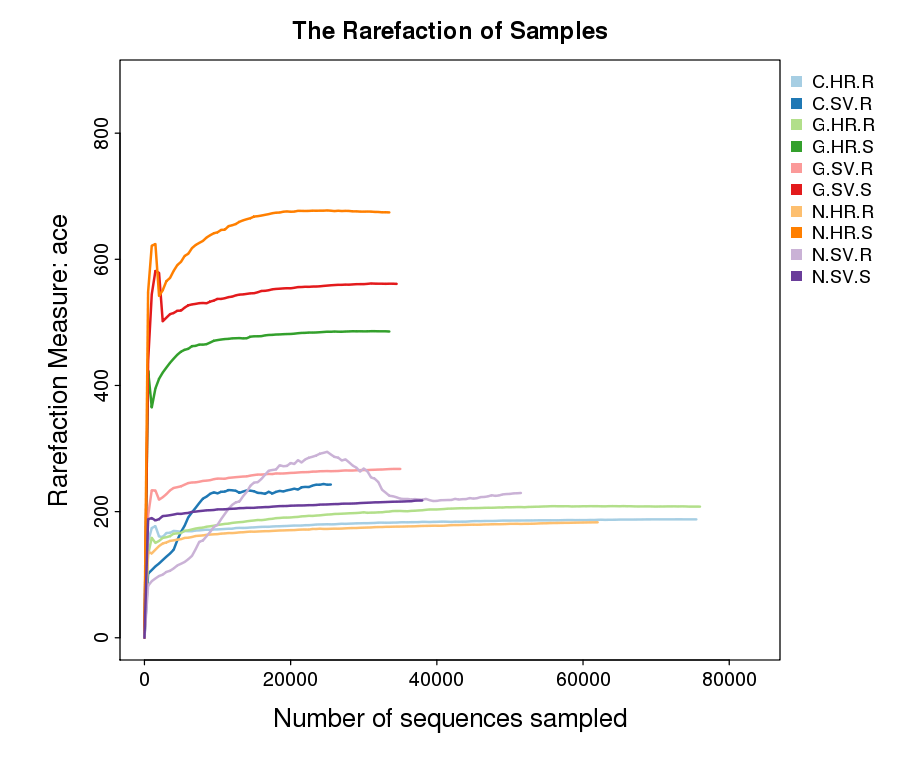


**Fig. S6**Alpha diversity of fungal communities indicating the biodiversity of the samples. A. Observed species, ACE; P < 0.05 B. Observed species, Chao; P < 0.05. Where, C: Cameroon; G: Gabon; N: Nigeria; HR: High rainfall; SV: Savannas


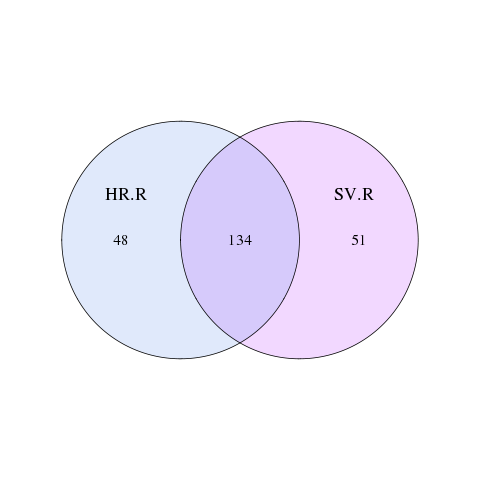

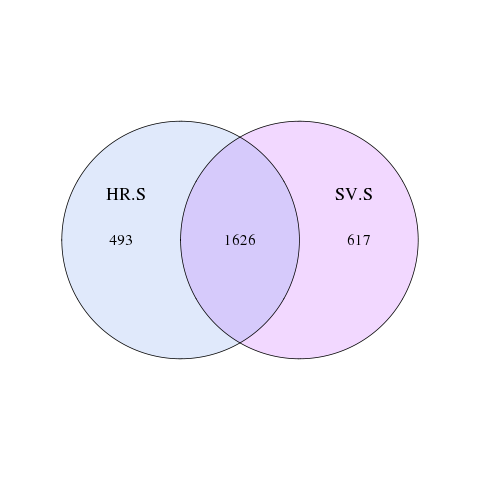

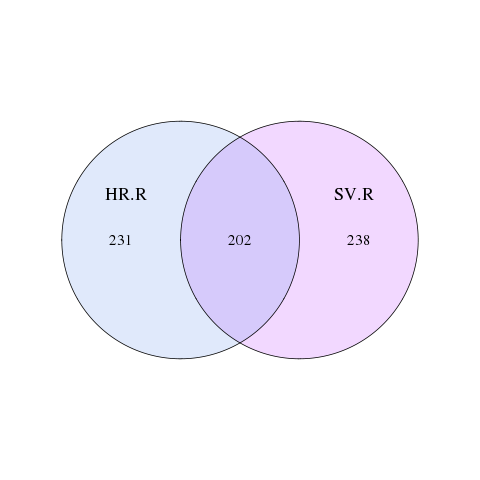

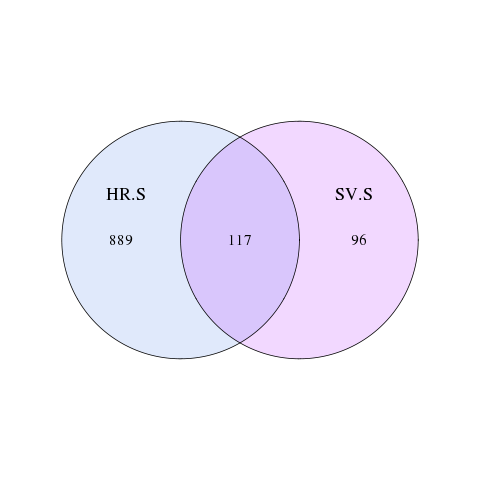


**Fig. S7**Venn diagram of the plantain rhizosphere. Up- bacterial and below- fungal community. The observed OTUs for each treatment were produced in the UCLUST algorithm to show the shared and unique OTUs. Only the most abundant OTUs among all the samples were represented. Where, HR: High rainfall; SV: Savannas; S: Rhizosphere soil; R: Plantain roots
